# Complete genome sequence of the probiotic *Lacticaseibacillus paracasei* LPC100 strain from the NORDBIOTIC collection isolated from a human fecal sample

**DOI:** 10.1128/mra.00344-24

**Published:** 2024-07-16

**Authors:** Agnieszka K. Szczepankowska, Bożena Cukrowska, Tamara Aleksandrzak-Piekarczyk

**Affiliations:** 1 Institute of Biochemistry and Biophysics, Polish Academy of Sciences, Warsaw, Poland; 2 Department of Pathomorphology, Immunology Laboratory, Children Memorial Health Institute, Warsaw, Poland; Loyola University Chicago, Chicago, Illinois, USA

**Keywords:** lactobacilli, probiotics, whole-genome sequencing, lactic acid bacteria, irritable bowel syndrome, bone mineralization, short-chain fatty acids, *Lacticaseibacillus paracasei*, fortification of intestinal barrier, iron adsorption

## Abstract

We report the genome sequence of the human fecal isolate *Lacticaseibacillus paracasei* LPC100 from the NORDBIOTIC collection, comprising a 3.075 Mb chromosome and three plasmids (61 kb, 12 kb, and 7 kb). Genetic content reveals the strain’s beneficial features—complete lactose metabolic pathway, potential production of bacteriocins, and short-chain fatty acids.

## ANNOUNCEMENT

*Lacticaseibacillus paracasei* is a gram-positive lactic acid bacterium. Individual *L. paracasei* strains carry probiotic properties beneficial for human health (1). *L. paracasei* LPC100, isolated from a healthy volunteer during a gut microbiome project, was acquired by Nordic Biotic Ltd., and deposited at the DSMZ collection (DSM33793). LPC100 is currently under clinical trial for its impact on bone mineral density in postmenopausal women and is a component of a multi-strain probiotic formulation for the treatment of irritable bowel syndrome (2).

The isolate was taken from NORDBIOTIC collection and cultured in MRS medium (Oxoid) (37°C, aerobic conditions). For genomic DNA isolation, bacterial cells were digested (15 min; 37°C) with lysozyme (20 mg/mL; Sigma-Aldrich) and mutanolysin (5 U/mL; A&A Biotechnology). DNA was extracted using the cetyltrimethylammonium bromide/lysozyme method (3) and sequenced by a hybrid approach using Illumina and Nanopore technologies. Illumina libraries were constructed using the NEB Ultra II FS kit (NEB) and sequenced in paired-end mode (v.3, 600 cycle chemistry kit) on a MiSeq instrument (Illumina). Sequence quality was assessed using FASTQC (v.0.12.0) (4), short reads were trimmed and filtered using fastp (v.0.23.2) (5), producing 485,740 paired reads and 203,757,345 nt of sequencing data.

For long-read library preparation, the same genomic DNA sample was sheared and fragments shorter than 10 kb were removed using the Short Read Eliminator kit (Circulomics). A 1D library was constructed using the SQK-LSK109 kit with the native barcoding expansion kit (EXP-NBD103) (ONT) and sequenced on a GridION sequencer using an R9.4.1 flowcell (ONT), producing 35,537 reads, 337,474,553 nt of sequence data with read length N_50_ of 14.6 kb.

Raw nanopore data were base-called using Guppy (v.6.1.3) (ONT) in high-accuracy mode. Quality filtering was done with NanoFilt (v.2.8.0) (6), adapters were removed by Porechop (v.0.2.4), and data set quality was checked with NanoPlot (v.1.41.6) (6). Data filtering resulted in 28,247 reads, and 334,125,171 nt of sequence data with a read length N_50_ of 14.8 kb.

Long-read assembly, circularization, and contig rotation were done using the Trycycler pipeline (v.0.5.3) (7). Nanopore reads were assembled with Flye (v.2.9), Unicycler (v.0.4.8), Raven (v.1.8.1), and Miniasm (0.3-r179). Trycycler consensus sequences were polished with Medaka (v.1.7.2). Long-read circular contigs were finally polished with Illumina data using Polypolish (v.0.5.0) (8) and POLCA (v.4.0.5) (9). Ambiguities were verified by Sanger sequencing using an ABI3730xl Genetic Analyzer and BigDye Terminator Mix (v.3.1) (ThermoFisher Scientific). Sequences were corrected using SeqMan software (DNAStar). Sequencing data are shown in Table 1.

**TABLE 1:** Sequence metrics of *Lacticaseibacillus paracasei* LPC100

Sequence ID	Size (bp)	%GC	CDS	Illumina coverage	ONT coverage	GenBank accession no.
LPC100 (chromosome)	3,075,670	46.27	2,902	56	76	CP143084
pLPC100_p1 (plasmid)	61,461	44.27	80	211	380	CP143085
pLPC100_p2 (plasmid)	11,830	40.76	11	701	5,722	CP143086
pLPC100_p3 (plasmid)	6,792	41.72	5	710	1,282	CP143087

Genome assembly revealed a single circular-closed chromosome and three circular plasmids. Annotation was done with the NCBI Prokaryotic Genome Annotation Pipeline (v.6.6) (10), predicting 2,998 coding sequences (CDSs), 59 tRNAs, 15 rRNAs, and 3 ncRNAs.

Gene searches identified four chromosomally encoded class IId potential bacteriocins, which may help to treat microbial dysbiosis (2). Complete lactose metabolism pathways were recognized, indicating that LPC100 is an efficient lactic acid producer with strong acidifying properties that can lower luminal pH and promote iron and calcium absorption (11 – 13). In addition, genes for short-chain fatty acid synthesis, which may fortify the intestinal barrier, and for peptidases capable of degrading peptides, including allergens, were identified (14).

## Data Availability

Complete sequencing data have been deposited in GenBank under BioProject: PRJNA1062316; BioSample: SAMN39212581. Whole-genomic data are available under accession no. CP143084, CP143085, CP143086, and CP143087. lllumina SRA reads are available under accession no. SRR27494225. Nanopore SRA reads are available under accession no. SRR27494226.
